# Observations on the Treatment of Several Diseases, as Practised in the General Hospital at Bamberg

**Published:** 1803-03-01

**Authors:** 


### *Observations on the Treatment of several Diseases, as practised in the General Hospital at Bamberg; by* Dr. Marcus. *Communicated by* Dr. Noehden.

DR. MARCUS, first physician to the celebrated hospital of Bamberg, made himself particularly conspicuous amongst the followers and well-wishers of the new system of medicine introduced by the principles of Dr. J. Brown. He endeavoured to show their consistency with practice and experience, by putting them to a trial at the bed of the sick patient; he has communicated the results of his observations in a periodical publication, which was intended, according to its title, to examine those principles of incitement by experiments made on the patient; and having ing been, by these means, convinced of the general consistency and practical utility of the new system, he thought proper to consider it, on the whole, as well founded, and consequently the farther continuation of this publication was no longer requisite. The present publication, however, may be taken for it, though appearing under a different title, and it contains the cases of diseases, as they occurred and were treated in the hospital at Bamberg.

In the first number, Dr. M. has premised some miscellaneous remarks, relating to the general treatment, as adopted in that hospital, which we consider, for more than one reason, as highly interesting to the practitioner.

"The only remedies which have for several years been employed in synochus as well as typhus, are opium, naphta, camphor, and musk; serpentaria and arnica are seldom used, and bark (cortex Peruvianus) is almost entirely exploded in the treatment of these diseases. The principal and most efficacious remedy however proves to be, according to our experience, opium in form of the most concentrated tincture. One of its chief effects in febrile diseases seems to originate in the property of producing costiveness more than any other known remedy. It is almost daily observed in our patients, that those who are for the longest time constipated, recover more readily than others. It is not uncommon with us to see patients afflicted with a typhus to remain costive for two, three, or four weeks; and even when they are in a state of re-convalescence, no alvine excretion takes place: But as soon as the bowels are once moved, the excretion will proceed as in the sound state, every twenty-four or forty-eight hours, even if the opium is continued. In patients, however, in whom the pressure of constipation seems to occasion several inconveniences, a clyster of an infusion of chamomile flowers and arnica is applied; but such cases are rarely observed. In putrid and nervous fevers, in which the head is particularly affected, and sopor and dizziness observable, symptoms which are generally considered to contraindicate the use of opium, it nevertheless proved of the greatest service, particularly in combination with warm aromatic fomentations. Besides opium, camphor was frequently employed in a dose of from two to five grains, in cases of synochus and typhus; sometimes it was alternately given with opium; and in case the patient disliked it, naphta was administered with equal success: but if the patient had any inclination to diarrhœa, naphta was discontinued, as being in this case not properly indicated.

The prejudicial effect of naphta in such cases, arises from its being impregnated with too much oxygen; and even if it is well prepared, it cannot be prevented, on opening the vessels in which it is contained, from entering into contact with the external air, and thus it becomes charged with oxygen."

Dr. Marcus could not conceive why a continual fever, or synochus, was frequently much sooner cured than a tertian fever, notwithstanding this originates in a far less degree of debility. For this singularity he could not account but by the manner of employing the volatile stimuli in both diseases. In the former they are generally given without intermission, often day and night; whereas in intermittent fevers, where danger is not so near, the treatment goes on more slowly, and the medicines are discontinued during the paroxysm and by night; by such spaces, however, the debility often increases as much as it has perhaps been diminished before by the administration of medicines. Induced by these and similar considerations, Dr. M. determined to treat intermittent fevers in the same manner as the continued ones, and he had the satisfaction to see his expectations crowned with success. All intermittent fevers treated in this manner were cured within a very few days.

In pulmonary consumption, opium likewise proved of the greatest efficacy; bark, lichen islandicus, senega, and digitalis were in all cases found to be either of no use at all, or at least very inefficacious remedies. In almost all cases a mixture of three drachms of myrrh, one drachm and a half of balsamus Peruvianus or canadensis, and half a drachm of extract. opii was of excellent service, it being made into pills of one grain each, and given alternately with tincture of opium, in a dose of two or three pills every hour. By this remedy the troublesome cough and expectoration were considerably diminished, proper rest procured, and the debilitating sweats and diarrhœas removed; it was likewise beneficial for the organs of digestion. By properly employing this remedy and adapting it to the degree of the disease, of the direct asthenia, and of the state of digestion, Dr. M. succeeded in curing patients, in whom every symptom of declared pulmonary consumption was observable.

In dropsies, little use has been experienced by those called diuretic medicines, which are, on the contrary, extremely hurtful, if not belonging to the class of incitants. But by properly attending to the degree of direct asthenia, in in which this disease originates; by adapting the plan of treatment to it, several very obstinate cases of dropsy have been radically cured, and in a proportionably short space of time. The remedies that were made use of, were either opium by itself, or in combination with liquid anodynes, essent. cort. aurant. or tinctura martis tonica.

"The most efficacious prescription in venereal complaints was a mixture of 3 ounces of aq. cinam. c. vino, four grains of corrosive sublimate, and two scruples of tinct. opii, of which from forty to eighty drops were to be given two or three times a day. Salivation has never been observed to follow the administration of this remedy, which even agreed with a weak constitution and digestion.

"In scabies we merely apply an unguent, consisting of three ounces of fat and one drachm of spir. nit. fumans; sometimes we also make use of the powder of charcoals. These remedies are quite sufficient, except in cases combined with a general disease, in which opium and warm baths are used. The external as well as internal use of sulphur is exploded from our practice in this disease. A series of experiments and observations has convinced us, that, besides mercury, there exists no specific remedy. The greatest and most useful specific is to explore the degree of the disease, and to adapt to it the choice and dose of the remedy.

"Ulcers are treated with us in a very simple manner; they are generally dressed dry; ointment and plasters are never made use of. In foul ulcers the dust of charcoal proved sometimes serviceable. For frictions, which however are but rarely applied, we formerly used to take opium; but being convinced of its inefficacy, when applied externally, it has been since disused, and instead of ointments, a mixture of one ounce of spirit. vin. rectificat. and one drachm of balsam. peruv. answered all our purposes in frictions.

"Amongst the persons wounded of the Gallo-Batavian army, the chief hospital of which was at Bamberg, in November, 1800, many were afflicted with tetanus; but Dr. Stutz's method of cure, employed with much care and accuracy, never answered our expectations. Opium in sufficient and increasing doses, immediately given, is the only and surest way of cure. Warm baths, prepared after Dr. Stutz's prescription, appear to be not so serviceable as those made of a decoction of aromatic herbs, mixed with a sufficient quantity of spirit of wine."

MONTHLY

